# Adipose Tissue: Understanding the Heterogeneity of Stem Cells for Regenerative Medicine

**DOI:** 10.3390/biom11070918

**Published:** 2021-06-22

**Authors:** Wee Kiat Ong, Smarajit Chakraborty, Shigeki Sugii

**Affiliations:** 1 School of Pharmacy, Monash University Malaysia, Subang Jaya 47500, Selangor, Malaysia; 2 Institute of Bioengineering and Bioimaging (IBB), A*STAR, 31 Biopolis Way, Singapore 138669, Singapore; schakraborty@ibb.a-star.edu.sg; 3 Cardiovascular and Metabolic Disorders Program, Duke-NUS Medical School, 8 College Road, Singapore 169857, Singapore

**Keywords:** adipose-derived mesenchymal stem/stromal cell (ASC, ADSC, AD-MSC), white adipose tissue (WAT), brown adipose tissue, beige adipose tissue, fat, adipocyte progenitor cell, ISCT and IFATS abbreviations, stromal vascular fraction (SVF), cell surface markers, cell therapy

## Abstract

Adipose-derived stem cells (ASCs) have been increasingly used as a versatile source of mesenchymal stem cells (MSCs) for diverse clinical investigations. However, their applications often become complicated due to heterogeneity arising from various factors. Cellular heterogeneity can occur due to: (i) nomenclature and criteria for definition; (ii) adipose tissue depots (e.g., subcutaneous fat, visceral fat) from which ASCs are isolated; (iii) donor and inter-subject variation (age, body mass index, gender, and disease state); (iv) species difference; and (v) study design (in vivo versus in vitro) and tools used (e.g., antibody isolation and culture conditions). There are also actual differences in resident cell types that exhibit ASC/MSC characteristics. Multilineage-differentiating stress-enduring (Muse) cells and dedifferentiated fat (DFAT) cells have been reported as an alternative or derivative source of ASCs for application in regenerative medicine. In this review, we discuss these factors that contribute to the heterogeneity of human ASCs in detail, and what should be taken into consideration for overcoming challenges associated with such heterogeneity in the clinical use of ASCs. Attempts to understand, define, and standardize cellular heterogeneity are important in supporting therapeutic strategies and regulatory considerations for the use of ASCs.

## 1. Types and Functions of Adipose Tissues

Adipose tissues play a pivotal physiological role in maintaining metabolic homeostasis in the body. White adipose tissue (WAT) stores excess energy in the form of triglyceride and is an endocrine organ that secretes adipokines. Adipocytes contain lipid droplets that store triglyceride, and they constitute approximately one third of the cells within adipose tissues [1]. Other cell types include adipose-derived stem cells (ASCs), preadipocytes, fibroblasts, endothelial cells, and immune cells [1,2]. ASCs become preadipocytes and subsequently differentiate into mature adipocytes via adipogenesis that itself involves activation of peroxisome proliferator-activated receptor gamma (PPARγ). Anatomically separated WAT depots, namely subcutaneous WAT (S-WAT) and visceral WAT (V-WAT), are known to be functionally distinct. S-WAT expands to store excess lipid, thus preventing ectopic lipid disposition and organ damage, while the main function of V-WAT is to cushion and protect the visceral organs [1,3]. On the other hand, brown adipose tissue (BAT), found in the cervical–supraclavicular region of the neck, perirenal/adrenal and paravertebral regions in adult humans, and the interscapular region in rodents [4,5], plays a significant role in thermogenesis via the actions of uncoupling protein 1 (UCP1). Interestingly, clusters of adipocytes within WAT can be induced via cold exposure or beta-adrenergic stimulation to become thermogenic beige adipocytes via a process described as “browning” [6].

## 2. Adipose Tissue as Source of Stem Cells

Contrary to the embryonic stem cells found in the inner cell mass of blastocyst, postnatal stem cells or adult stem cells, such as mesenchymal stem cells (MSCs), can be found in almost all postnatal organs and tissues, most notably in bone marrow, WAT, amniotic fluid, dental tissues, blood, placenta, skin, synovial fluid, and Wharton’s jelly [7,8]. The discovery of adult stem cells in WAT is of biological and clinical significance. The first characterization of these cells, isolated from the human lipoaspirates, was reported about two decades ago in 2002 [9,10]. These adult stem cells, which Zuk et al. named as processed lipoaspirate (PLA) cells, are ASCs that exhibit the properties of MSCs [9]. The high abundance of ASCs in the WAT [11] and easy accessibility of S-WAT beneath the skin are obvious advantages of S-WAT over conventional bone marrow and other tissues, especially when it comes to a reliable, safe, and feasible source of MSCs for tissue engineering and regenerative medicine [7]. The focus of this review will be on human adipose tissue as a source of stem cells that meet the current definition of MSCs.

## 3. Terminology and Definition

By the convention of the International Society for Cell and Gene Therapy (ISCT) in 2006, the abbreviation “MSCs” should be used for multipotent “mesenchymal stromal cells” that are (i) plastic-adherent in the standard cell culture condition, (ii) able to demonstrate trilineage mesenchymal differentiation into osteoblasts, adipocytes, and chondrocytes in vitro, and (iii) CD73^+^, CD90^+^, CD105^+^, CD11b^−^ or CD14^−^, CD19^−^ or CD79α^−^, CD34^−^, CD45^−^, and HLA-DR^−^ in their cell surface immunophenotype [12]. The ISCT reiterates that the term “mesenchymal stem cells” should only be used if there is rigorous functional evidence in vitro and in vivo to demonstrate the stemness of the isolated cells, namely the ability to self-renew or proliferate and differentiate [13].

Names and abbreviations that have been used for the MSCs isolated from adipose tissue include PLAs, adipose-derived adult stem (ADAS) cells, adipose-derived stromal cells (ADSCs), adipose mesenchymal stem cells (AdMSCs), and lipoblasts. To address the confusion over terminology, the International Federation for Adipose Therapeutics and Science (IFATS) has proposed “adipose-derived stem cells (ASCs)” as the standard nomenclature [14]. In 2019, the ISCT suggested the term “adipose tissue-derived MSCs” (AD-MSCs), thus recommending that the tissue-source origin should be a part of the MSC nomenclature [13]. The term “ASCs” is used in this review.

The revised statement published by the IFATS and the ISCT in 2013 proposed that the freshly isolated uncultured adipose stromal cell population, containing native ASCs, is characterized as CD45^−^, CD235a^−^, CD31^−^, and CD34^+^ cells. Additional positive markers that can be considered for characterization purposes are CD13, CD73, CD90, CD105, CD10, CD29, and CD49 [2]. After culture, ASCs are CD45^−^, CD31^−^, CD73^+^, CD90^+^, CD105^+^ and/or CD13^+^, and CD44^+^. Suggested additional positive markers are CD10, CD26, CD49d, and CD49e, while low or negative markers include CD3, CD11b, CD49f, CD106, and podocalyxin-like protein (PODXL). In contrast to bone-marrow-derived MSCs (BM-MSCs) that are CD36^−^ and CD106^+^, cultured ASCs are CD36^+^ and CD106^−^ [2]. The cell surface immunophenotype should be coupled with a proliferation assay (e.g., a fibroblastoid colony-forming unit (CFU-F) assay) and trilineage mesenchymal differentiation assays to complete cell identification [2].

It is noteworthy that, in 2006, CD34 was specified as a negative marker for MSCs by the ISCT convention [12]. Bourin et al. recommend the use of class III CD34 antibodies for cell surface immunophenotype characterization [2]. It is known that the expression of CD34 is dependent on the culture condition, donor, and passage [2,7,13]. A systematic review by Mildmay-White and Khan shows that ASCs from adult humans are commonly reported to be CD90^+^, CD44^+^, CD29^+^, CD105^+^, CD13^+^, CD34^+^, CD73^+^, CD166^+^, CD10^+^, CD49c^+^, CD59^+^ and CD31disagreements over ^−^, CD45^−^, CD14^−^, CD11b^−^, CD34^−^, CD19^−^, CD56^−^, and CD146^−^, with the expression of CD31, CD34, CD117, and STRO-1 [15].

## 4. Stromal Vascular Fraction (SVF)

S-WATs are commonly harvested by Coleman’s technique (manual harvest of fat aspirated with a blunt cannula and a syringe), machine-assisted liposuction, and surgical resection. The choice of harvesting techniques affects cell viability and consequently the yield of ASCs [16,17]. The harvested WAT can be used with minimal processing for autologous fat grafting in aesthetic and reconstructive procedures, which include correction of contour abnormalities, breast reconstruction, and cosmetic procedures [17,18]. Alternatively, WAT may be further processed for ASC isolation.

**Figure 1:**
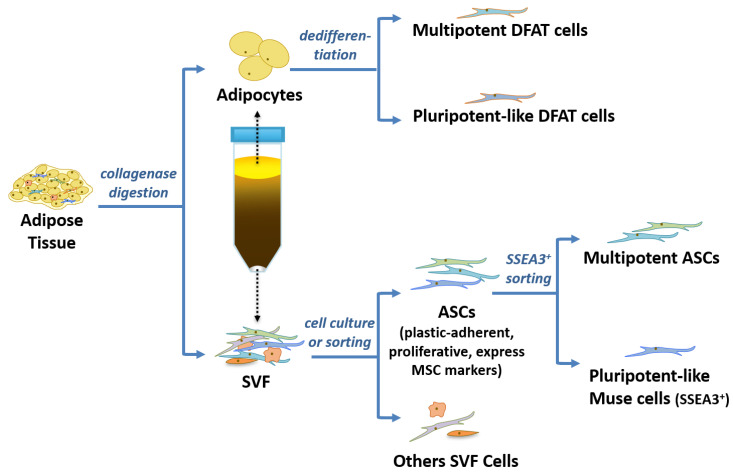
Adipose tissue is a source of diverse cell types for application in regenerative medicine. Adipocytes are separated from the stromal vascular fraction (SVF) by collagenase digestion and centrifugation. Adipose-derived stem cells (ASCs) expanded in culture display characteristics of mesenchymal stem cells (MSCs) but are still heterogeneous (Section 6 and Section 7). Sorting of ASCs by SSEA3 expression isolates multilineage-differentiating stress-enduring (Muse) cells (Section 8). Dedifferentiated fat (DFAT) cells can be generated from floating adipocytes using ceiling culture or cell filter methods (Section 9).

The first step in the isolation of ASCs from WAT involves the separation of adipocytes from the remaining adipose cells of the SVF (Figure 1). This is typically achieved by collagenase digestion of WAT and centrifugation to separate the floating adipocytes from the pelleted SVF [19]. To comply with current good manufacturing practices (cGMPs), closed, sterile, and safe isolation processes have been developed. They can involve enzymatic or mechanical procedures to release the cellular components using an automated closed device [20] or a cost-effective protocol alternative to automated methods [21].

The SVF is a mixed population of stromal cells (comprising ASCs, preadipocytes, and fibroblasts), CD45^+^ hematopoietic-lineage cells (comprising hematopoietic stem and progenitor cells, granulocytes, lymphocytes, monocytes/macrophages, and CD45^−^ CD235a^+^ erythrocytes), CD146^+^ pericytes, CD31^+^ endothelial cells, and smooth muscle cells [2]. Freshly isolated SVF can be used directly without the need for further cell separation and in vitro expansion. This has been shown to be advantageous due to the desired synergistic contribution by the various stromal vascular components. That said, they are known to cause immunological rejection and therefore restrict SVF to use in mainly autologous treatments [22,23].

ASCs constitute as much as 1% of SVF cells compared with the 0.001–0.002% of BM-MSCs in bone marrow [11]. Erythrocytes are usually removed using a lysis buffer with the standard protocol [19]. ASCs in the SVF can be selectively enriched by plastic adherence incubation, immunomagnetic separation or flow cytometry cell sorting, and in vitro cell culture expansion. However, isolation of ASCs can be hampered by challenges in separating ASCs from other cell types that have a similar cell surface immunophenotype and similar cell culture properties, notably preadipocytes and fibroblasts. The removal of contaminating preadipocytes and fibroblasts prevents the dilution or loss of ASCs in the culture for use in regenerative medicine.

The unipotent PPARγ-expressing preadipocytes are CD54^+^ in human S-WAT and committed to differentiation into adipocytes [24]. The multipotent CD26^+^ ASCs within the SVF are highly proliferative [24] and enriched through cell culture passaging. Fibroblasts are often co-isolated from the WAT and they can rapidly overgrow ASCs in culture. The two cell types share a spindle-like morphology when they adhere to plastic. Fibroblasts have been reported to express the putative ISCT MSC surface markers [25,26,27]. Alt et al. reported that fibroblasts lack the differentiation and colony-forming potential of MSCs, despite them expressing the MSC cell surface immunophenotype [28], whereas the study by Denu et al. showed that fibroblasts are phenotypically indistinguishable from MSCs in terms of differentiation potential and immunoregulatory properties [26]. Fibroblasts have even been proposed to be practical alternatives to MSCs in regenerative medicine due to the shared properties between the two cell types [29]. In the ongoing search for molecular signatures that distinguish fibroblasts from ASCs or MSCs in general, novel surface markers, e.g., transmembrane 4 L6 family member 1 (TM4SF1), CD146, CD166 (predominantly expressed on MSCs) [27,30,31], and CD9 (predominantly expressed on fibroblasts) [31], the epigenetic signature [32], and the secretion profile of specific growth factors [33] have been proposed.

## 5. ASCs in Regenerative Medicine

In regenerative medicine, autologous or allogeneic ASCs have been employed in clinical trials to treat conditions such as lipoatrophy, muscular dystrophy, liver cirrhosis, myocardial infarction, stroke, spinal cord injury, graft-versus-host disease (GVHD), osteoarthritis, Crohn’s disease, and cancer [34,35]. Like other MSCs, ASCs can differentiate into cells of mesodermal (osteoblasts, adipocytes, and chondrocytes), endodermal (hepatocytes, pancreatic β cells), and ectodermal (neurons) origin upon in vitro induction [36]. Differentiation of ASCs to the specialized cells of interest enables the replacement of damaged, diseased, and defective cells and tissues. In addition, ASCs secrete an array of angiogenic, anti-apoptotic, and hematopoietic factors that facilitate tissue repair and regeneration via autocrine and paracrine actions. ASCs also secrete anti-inflammatory or immunosuppressive factors and exert immunomodulatory effects in the cell therapy of GVHD, autoimmune diseases, and inflammatory diseases [35,37]. ASCs release exosomes and other extracellular vesicles that carry bioactive cargo (such as proteins, lipids, DNA, mRNA, micro-RNA, tRNA, and noncoding RNA) with immunomodulatory and regenerative properties [38,39]. ASC-derived exosomes and conditioned media are promising cell-free therapeutic approaches in regenerative medicine [38]. To further enhance ASC function for use in regenerative medicine, ASCs are also pre-conditioned with bioactive molecules, genetically modified, and grown in three-dimensional aggregates and hypoxic culture [40].

A major hurdle in the evaluation of pre-clinical studies, clinical trials, and eventual clinical translation is the lack of standardization of ASCs. Cellular heterogeneity can occur due to: (i) nomenclature and criteria for definition, which have been largely standardized by the IFATS and the ISCT; however, a more precise definition with a specific molecular signature is still needed; (ii) the adipose tissue depot (e.g., S-WAT versus V-WAT) from which ASCs are isolated; (iii) donor and inter-subject variation (age, body mass index, gender, and disease state) [41]; (iv) species difference; and (v) study design (in vivo versus in vitro) and tools used (e.g., antibody isolation and culture conditions).

## 6. ASCs from Separate Adipose Tissue Depots

It is widely recognized that ASCs that meet the current ISCT criteria for MSCs are still heterogeneous in function and immunophenotype despite expressing the putative MSC cell surface markers. These biological properties of ASCs are dependent on the source of adipose tissue from where they are derived, namely the anatomical location of the adipose depot within a human subject. Depot-dependent cellular difference can be attributed to the inherent diversity of ASCs alongside epigenetic memory, which is due to the variable cellular composition and microenvironment in adipose tissues across separate depots. This depot-dependent cellular difference is retained in expanded ASCs in cell culture conditions. The choice of adipose depot as the source of ASCs can be determined by the intended application in regenerative medicine.

The S-WAT is the most common and clinically practical source of ASCs. Iwen et al. compared subcutaneous ASCs (S-ASCs) isolated from gluteal and abdominal S-WATs and showed that gluteal S-ASCs exhibited higher adipogenic and osteogenic differentiation potential than abdominal S-ASCs [42]. Interestingly, di Taranto et al. found that SVF and S-ASCs isolated from the superficial layer—which is separated by superficial fascia from the deep layer of abdominal S-WAT—exhibited higher viability, stemness marker expression, and adipogenic and osteogenic potential than those from the deep layer [43].

Studies that compared S-ASCs with donor-matched visceral ASCs (V-ASCs) isolated from omental [44,45,46] and retroperitoneal [47] V-WAT depots reported depot-dependent variability in the yield, viability, immunophenotype, proliferation, differentiation potential, secretome, and gene expression profiles of the ASC populations. The S-WAT and V-WAT have different roles in metabolic homeostasis [3,6] and differ in their developmental origins [48,49]. Isolated ASCs retain inherent depot-dependent cellular characteristics. Kim et al. reported upregulated clusters of genes related to lipid biosynthesis and metabolism in retroperitoneal V-ASCs, whereas abdominal S-ASCs highly expressed genes relevant to DNA-dependent transcription, and thus contributed to proliferation [47]. A notable functional difference is that cultured abdominal S-ASCs differentiate better than omental and retroperitoneal V-ASCs in response to in vitro adipogenic stimuli [44,45,47]. These ASCs also exhibit a depot-dependent cell surface immunophenotype with a predominant expression of CD10 and CD200 in the abdominal S-ASCs and omental V-ASCs, respectively [44]. CD10 expression correlated positively with ASC adipogenic potential; this is consistent with the notion that the S-WAT can effectively increase its lipid-storing capacity by hyperplasia of adipocytes [44,50]. Being high in CD200 expression, omental V-ASCs differentiate better than S-ASCs in response to in vitro osteogenic induction, suggesting that CD200 can be a potential osteogenic marker that regulates osteogenesis [44,45]. This is consistent with the finding by Kim et al. that reported higher osteogenesis in BM-MSCs transfected with CD200 [51]. V-WAT expansion underlying pathological obesity is associated with inflammation [3]. At the cellular level, omental V-ASCs secrete higher levels of inflammatory cytokines, such as interleukin-6, interleukin-8, and tumor necrosis factor α, relative to abdominal S-ASCs [46]. In addition, S-ASCs and omental V-ASCs are also known to vary in their exosome contents [52].

ASCs have also been isolated from non-conventional depots, such as mediastinal [53] and pericardial adipose tissue in the intrathoracic compartment [45] and perirenal adipose tissue in the visceral compartment [54]. These adipose tissues resemble WAT in appearance but possess the characteristics of beige or BAT [5,39,55]. Jespersen et al. reported widespread amounts of dormant BAT, identifiable by multilocular brown adipocytes, among WAT throughout the perirenal depot, especially near the adrenal gland [55]. For example, pericardial ASCs differentiate better than omental V-ASCs in response to in vitro adipogenic stimuli [45]. Silva et al. demonstrated that mediastinal ASCs expressed the putative MSC surface markers and were capable of trilineage mesenchymal differentiation. However, they also expressed BAT-specific genes, such as PRDM16, UCP1, IRS2, and NRF1, and differentiated into metabolically active brown adipocytes that could potentially be used for the treatment of obesity and related metabolic disorders [53]. From a clinical translational perspective, the potential use of these ASCs is restricted by the limited availability and accessibility of the relevant adipose tissue depots. ASCs have also been isolated from solitary subcutaneous lipoma, a benign adipose tissue tumor, and they show putative MSC phenotypes [56].

## 7. ASCs from the Same Adipose Tissue Depot

Cellular heterogeneity exists even among the ASCs isolated from the same adipose depot. Cultured ASCs from the same source can be further sorted by cell surface immunophenotype into subpopulations with a distinct differentiation potential into the cell type of interest [57]. Interestingly, González-Cruz and Darling demonstrated that an immunolabel-free approach could be employed to sort ASCs into subpopulations with a variable propensity to differentiate into adipocytes, chondrocytes, and osteoblasts, based on the elastic and viscoelastic properties of ASCs [58]. The presence of ASC subpopulations may be due to cells with distinct inherent characteristics and developmental origin, as well as a temporal stem cell phenotype at different stages of growth and acquired variation due to isolation procedures and culture conditions in vitro.

The developmental origin of ASCs is still unclear, but at least a subset of ASCs originate from CD146^+^, neuro-glial proteoglycan 2 (NG2)^+^, CD140β^+^ pericytes, or vascular precursor cells within the blood vessels [7,59]. Analysis of single-cell-derived ASC clones revealed that 81% of the clones differentiated into at least one of the following cell types: osteoblasts (48%), chondrocytes (43%), adipocytes (12%), and neuron-like cells (52%) at passage 4, highlighting the presence of inherently diverse subpopulations [60]. Single-cell RNA sequencing (scRNAseq) identified a CD26^+^ CD55^+^ ASC subpopulation that displayed enhanced cell survival, stemness, proliferative, and colony-forming potential as compared with the unsorted parent ASC population [61]. A subpopulation within omental V-ASCs with characteristics of beige preadipocytes has also been uncovered by scRNAseq [62].

Isolated SVF containing ASCs is commonly maintained in an adherent in vitro cell culture for at least two weeks, with two or three passagings at 80% confluence to enrich and expand ASCs with putative MSC phenotypes [19,63]. A significant difference in differentiation potential, secretome, and gene expression has been reported between ASCs enriched by cell culture and an uncultured ex vivo ASC population freshly isolated from SVF by immunomagnetic separation [63]. Culture duration and passage number can result in selective enrichment of certain subpopulations and/or a time-dependent phenotypic change in ASCs, as is evident from the dynamic change in cell surface immunophenotype with passaging [7,64]. Iminitoff et al. reported an increased expression of miR-31, which is a general inhibitor of differentiation, and a decreased expression of pro-adipogenic miR-378 with culture duration [65]. ASCs at higher passage numbers are associated with senescence, decreased proliferation and differentiation potential [63,66], as well as an increased incidence of genetic abnormalities [67]. Jeske et al. found that ASCs were less susceptible to culture stress relative to BM-MSCs. An increased secretion of pro-inflammatory cytokines and decreased secretion of anti-inflammatory cytokines was observed after extended culture expansion of ASCs [68].

There is no standardized isolation procedure and culture protocol for ASC expansion. As reviewed by Baer and Geiger, the variables in culture conditions due to the difference in basal medium composition, glucose concentration, serum and growth factors, oxygen supply, coating of culture dishes, cell density, subculturing method, and cryopreservation could have a significant impact on the phenotype of the ASCs in vitro [7,69]. Ahearne et al. reported that basal media with fibroblast growth factor 2 (FGF2) decreased adipogenic potential, while high-glucose basal media with FGF2 increased the osteogenic potential of S-ASCs at passage 4. Supplementation of basal media with FGF2 promoted chondrogenesis [70]. ASCs are conventionally cultured under normoxic conditions (21% O_2_), but oxygen levels within tissue are generally much lower. ASCs cultured under hypoxic culture conditions (1–2% O_2_), which mimic the in vivo environment, have been reported to exhibit reduced CD105 expression [71], an increased proliferation rate [72], and increased adipogenic [73], chondrogenic [72], and osteogenic potential [73,74] compared with ASCs cultured under normoxic conditions.

## 8. Multilineage-Differentiating Stress-Enduring (Muse) Cells

Muse cells were initially isolated from BM-MSCs, bone marrow aspirates, skin fibroblasts [75], and adipose tissue [76] through the application of severe cellular stress, namely long-term exposure to trypsin or collagenase, serum deprivation, low temperatures, and hypoxia; hence, Muse cells are described as stress-enduring. It is known that Muse cells can be isolated from cultured ASCs based on the positive expression of embryonic stem cell surface marker SSEA3 and MSC marker CD105 [77,78]. The CD90^+^, CD105^+^, and SSEA3^+^ Muse cells accounted for 3.8–8.8% of the cultured ASCs [77]. Muse cells form characteristic cell clusters similar in morphology to embryonic stem cell-derived embryoid bodies in suspension culture and cell aggregates when they adhere to the dish [76,77,79]. Muse cells express pluripotency markers (SSEA3, Oct3/4, Nanog, and Sox2) and can undergo triploblastic differentiation into ectodermal, endodermal, and mesodermal cell lineages; however, they are non-tumorigenic, as they do not form teratoma in vivo [76,77]. There has been evidence to support the presence of endogenous Muse cells that can be mobilized from bone marrow to the peripheral blood circulation. These Muse cells act as endogenous reparative stem cells that sense and migrate to the location of the damaged tissue to exert functional and structural repair via immunomodulatory, anti-apoptotic, anti-fibrotic, and angiogenic effects, as well as by spontaneous differentiation into tissue-compatible cells after the homing of the Muse cells to the damaged tissue [80].

As they are non-tumorigenic, Muse cells have an advantageous safety profile and are a subpopulation of MSCs that include ASCs, which are already widely applied in clinical trials. In addition, Muse cells are inherently pluripotent-like and are capable of cell homing damaged tissue following intravenous injection. Due to these unique properties, Muse cells have been explored as a potential therapy to treat acute myocardial infarction, stroke, chronic kidney disease, liver diseases, and neurologic diseases [81,82].

## 9. Dedifferentiated Fat (DFAT) Cells

Contrary to the previous notion that adipogenesis is a terminal differentiation process, it is also known that, with the loss of lipid droplets, adipocytes can dedifferentiate to become fibroblast-like cells, also known as DFAT cells [83]. As reviewed by Song and Kuang, evidence from animal models indicates that physiological dedifferentiation of adipocytes occurs in vivo during lactation in the mammary gland. They add that pathological adipocyte dedifferentiation has been implicated in conditions such as cutaneous fibrosis, wound healing, and cancers [84]. This suggests that a subpopulation of ASCs may be in vivo DFAT cells.

The most common culture method to generate DFAT cells in vitro from isolated adipocytes is the ceiling culture method. This involves dedifferentiation of floating adipocytes that attach to the top inner surface or ceiling of a fully filled flask, which is then inverted after 7 days of incubation [85,86]. DFAT cells express putative MSC surface markers CD13, CD29, CD44, CD49d, CD73, CD90, and CD105, and do not express CD11b, CD14, CD31, CD34, CD45, CD106, or HLA-DR [86,87]. Saler et al. reported that the cell surface immunophenotype of DFAT cells and ASCs at passage 0 was essentially the same. DFAT cells were comparable to ASCs in terms of proliferation and capable of trilineage mesenchymal differentiation [87]. DFAT cells have been generated from S-WAT [86,87] and the buccal fat pad [88,89]. Tsurumachi et al. compared DFAT generated from small adipocytes (cell diameters less than 40 μm) and large adipocytes (cell diameters of 40–100 μm) in the buccal fat pad, and found that the small adipocytes dedifferentiated better into DFAT cells compared with the large adipocytes. The DFAT cells generated from the small adipocytes also contained a larger proportion of CD146^+^ cells and exhibited higher osteogenic potential [88]. DFAT cells were reported to be relatively homogenous compared with ASCs. This could translate to higher predictability in terms of safety and efficacy when used in regenerative medicine [86,89].

Jumabay et al. developed a novel method to generate DFAT cells by culturing isolated adipocytes in a six-well plate fitted with 70 mm filters and incubated for 5 days. DFAT cells generated from the floating adipocytes then pass through the filter and attach to the bottom of the plate [90]. Interestingly, they found that the early DFAT cells, those present 5–7 days post adipocyte isolation, expressed pluripotency markers (SSEA3, SSEA4, Oct3/4, Nanog, SOX2, Klf4, and c-Myc) alongside CD105 and formed cell aggregates in culture [90]. The DFAT cells spontaneously differentiated in a basic medium with a time-dependent decrease in pluripotency marker expression and a corresponding increase in the expression of lineage markers from the three germ layers, suggesting that the early DFAT cells were capable of triploblastic differentiation. When maintained in a defined medium that deterred differentiation, the DFAT cells did not form teratoma in vivo [90,91].

## 10. Future Perspective

Adipose tissue is an accessible source of heterogeneous populations of ASCs, as well as Muse cells and DFAT cells, for application in regenerative medicine (Table 1). As discussed earlier, the properties of these cells vary depending on where they are isolated from, how they are expanded, and when they are studied. Adipose-derived non-tumorigenic pluripotent-like stem cells with extended multipotency have also been reported. An improved understanding of factors underlying cellular heterogeneity and phenotypic implications enables appropriate subpopulations to be sourced and expanded for a customized and targeted approach that depends on the treatment objectives in regenerative medicine. Standardization of cell processing and culture protocols needs to be in place for better evaluation of pre-clinical studies and clinical trials with respect to the efficacy and safety of potential therapeutic applications. The validation and establishment of standard protocols for clinical-grade, cGMP-compliant stem cells and their derivatives on a scalable bioprocessing platform ensures the quality of the cells for translational use.

## Figures and Tables

**Table 1:** Characteristics of cultured adipose-derived cells from representative studies.

Cell Types	Source of Cells	Isolation and Culture Conditions	Cell Surface Immunophenotype	Differentiation Potential and Other Characteristics	References
MSC (by definition)	Bone marrow and other tissues including adipose tissues	-	CD73^+^, CD90^+^, CD105^+^, CD11b^−^ or CD14^−^, CD19^−^ or CD79α^−^, CD34^−^, CD45^−^, and HLA-DR^−^	Multipotent (trilineage mesenchymal differentiation into osteoblasts, adipocytes, and chondrocytes in vitro). Plastic-adherent in the standard cell culture condition. Evidence of ability to proliferate and differentiate to be termed “stem cells”.	ISCT [12,13]
ASC (by definition)	Adipose tissues	-	CD45^−^, CD31^−^, CD73^+^, CD90^+^, CD105^+^ and/or CD13^+^, and CD44^+^. Other positive markers: CD10, CD26, CD49d, and CD49e. Low or negative markers: CD3, CD11b, CD49f, CD106, and PODXL. Unlike BM-MSCs, ASCs are CD36^+^ and CD106^−^.	Multipotent (trilineage mesenchymal differentiation). Proliferation potential (CFU-F assay).	IFATS and ISCT [2]
S-ASC versus V-ASC	Abdominal S-WAT versus Omental V-WAT	Dulbecco’s modified Eagle’s medium (DMEM) high glucose with 15% fetal bovine serum (FBS) and 5 ng/mL FGF2	Both S-ASCs and V-ASCs: CD73^+^, CD90^+^, CD105^+^. S-ASCs: high CD10 expression. V-ASCs: high CD200 expression.	Both S-ASCs and V-ASCs: capable of trilineage mesenchymal differentiation. S-ASCs differentiated better than V-ASCs in response to adipogenic stimuli.	[44]
Mediastinal ASC	Mediastinal adipose tissue	DMEM low glucose with 10% XcytePlus	CD73^+^, CD90^+^, CD105^+^, SSEA4^+^, 72% CD137^+^	Capable of trilineage mesenchymal differentiation. Expressed BAT-specific genes, such as PRDM16, UCP1, IRS2, and NRF1, and differentiated into metabolically active brown adipocytes.	[53]
Adipose Muse cell	S-WAT	Adipose Muse cells were isolated by sorting of SSES3^+^ CD105^+^ S-ASCs maintained in DMEM high glucose with 15% FBS. Adipose Muse cells were then cultured in suspension in alpha-MEM with 15% FBS.	SSES3^+^, CD105^+^, CD90^+^, CD34^−^, CD146^−^	Formed cell clusters in single-cell suspension culture, expressed pluripotency markers (Nanog, Oct3/4, PAR4, Sox2, and TRA-1-81). After the cell clusters were transferred into a gelatin-coated dish, expanded cells differentiated spontaneously and were positive for markers for the three germ layers. No teratoma formation in vivo.	[77]
DFAT cell versus S-ASC	S-WAT from the peritro-chanteric region	DFAT cells were generated by the ceiling culture method in DMEM F12-HAM with 20% FBS DFAT cells and S-ASCs were then cultured in DMEM F12-HAM with 10% FBS.	Both DFAT cells and S-ASCs: CD13^+^, CD73^+^, CD90^+^, CD105^+^, CD14^−^, CD34^−^, CD45^−^.	Both DFAT cells and S-ASCs: capable of trilineage mesenchymal differentiation, similar proliferative potential.	[87]
DFAT cell	S-WAT	DFAT cells, generated from the floating adipocytes within 5 days post-isolation, sank through a cell filter to the bottom of the plate. DFAT cells were cultured in DMEM with 20% FBS.	Early DFAT cells (5–7 days post adipocyte isolation): CD105^+^, SSEA3^+^, SSEA4^+^.	Formed cell aggregates in culture, expressed pluripotency markers (Oct3/4, Nanog, SOX2, Klf4, and c-Myc). DFAT cells spontaneously differentiated in a basic medium and were positive for markers of the three germ layers. No teratoma formation in vivo.	[90]

MSC, mesenchymal stem cell; ASC, adipose-derived stem cell; BM-MSC, bone-marrow-derived MSC; CFU-F, fibroblastoid colony-forming unit; S-ASC, subcutaneous ASC; V-ASC, visceral ASC; S-WAT, subcutaneous white adipose tissue; V-WAT, visceral white adipose tissue; FGF2, fibroblast growth factor 2; Muse cell, multilineage-differentiating stress-enduring cell; DFAT cell, dedifferentiated fat cell.

## Data Availability

Not applicable.
